# Branched-Chain Amino Acids Ameliorate Fibrosis and Suppress Tumor Growth in a Rat Model of Hepatocellular Carcinoma with Liver Cirrhosis

**DOI:** 10.1371/journal.pone.0077899

**Published:** 2013-11-01

**Authors:** Jung Hoon Cha, Si Hyun Bae, Hye Lim Kim, Na Ri Park, Eun Suk Choi, Eun Sun Jung, Jong Young Choi, Seung Kew Yoon

**Affiliations:** 1 The Catholic University Liver Research Center, The Catholic University of Korea, Seoul, Republic of Korea; 2 Department of Internal Medicine College of Medicine, The Catholic University of Korea, Seoul, Republic of Korea; 3 Hospital Pathology, The Catholic University of Korea, Seoul, Republic of Korea; Yonsei University College of Medicine, Republic of Korea

## Abstract

**Purpose:**

Recent studies have revealed that branched-chain amino acids (BCAA) reduce the development of hepatocellular carcinoma (HCC) in patients with obesity and hepatitis C virus infection by improving insulin resistance (IR). The aim of this study was to examine the anti-cancer and anti-fibrotic effects of BCAA on the development of diethylnitrosamine (DEN)-induced HCC and liver cirrhosis in a rat model.

**Methods:**

Male SD rats received weekly intraperitoneal injections of DEN (50 mg/kg of body weight) for 16 weeks to induce HCC. They were fed a diet containing 3% casein, 3% or 6% BCAA for 13 weeks beginning 6 weeks after DEN administration. DEN was used to induce HCC through stepwise development from cirrhosis to HCC. The effect of BCAA was evaluated in tumor tissues by histopathologic analyses, reverse transcription-polymerase chain reaction, and Western blotting.

**Results:**

The mean area and number of dysplastic nodules (DNs) and tumors in the casein group tended to be larger than those in the BCAA group 16 weeks after DEN administration. The mean fibrotic area in the BCAA group was smaller than that in the casein group. The BCAA group showed decreased mRNA levels for markers of fibrosis, angiogenesis, and apoptosis inhibition. Compared with the casein group, the BCAA group had lower levels of α-smooth muscle actin, vascular endothelial growth factor, p-β-catenin, p-p38 mitogen-activated protein kinase, proliferating cell nuclear antigen, and caspase-3 protein expression, as well as a higher level of cleaved caspase-3 protein expression.

**Conclusions:**

BCAA supplementation of the diet ameliorated liver fibrosis and HCC development in a DEN-induced rat model of HCC with liver cirrhosis, but not in the IR model. These results provide a rationale for anti-fibrosis and chemoprevention using BCAA treatment for HCC with liver cirrhosis, as well as decreasing the ammonia level.

## Introduction

Hepatocellular carcinoma (HCC) is the fifth most common cancer and the third leading cause of cancer death worldwide [1]. Development of HCC is commonly associated with chronic inflammation of the liver induced by ongoing viral hepatitis and cirrhosis [2]. The risk of HCC is higher in persons who are obese and have diabetes mellitus, which is associated with hyperinsulinemia [3]–[7].

Nutritional supplementation with oral branched-chain amino acids (BCAA; leucine, isoleucine, and valine) is useful for improving protein malnutrition, increasing the serum albumin concentration, slowing the progression of hepatic failure, and prolonging event-free survival in patients with liver cirrhosis [8]–[11]. Furthermore, several studies have revealed that BCAA reduces the risk of obesity- and hepatitis C virus-related HCC by improving insulin resistance (IR) [12]–[16]. However, the anti-fibrotic and anti-cancer mechanisms of BCAA remain to be elucidated.

Transforming growth factor-β1 (TGF-β1) plays a key role in the pathogenesis of liver fibrosis by inducing fibroblasts to synthesize and contract the extracellular matrix (ECM) [17]. This cytokine also induces collagen formation by increasing procollagen mRNA levels [18]. Furthermore, Smad-4 promotes fibrosis as a downstream mediator of TGF-β1, and hepatic fibrosis results from the inhibition of ECM degradation through specific tissue inhibitors of metalloproteinases [19], [20].

Angiogenesis plays an important role in tumor growth [21]–[23]. Angiogenesis is a particularly important process in HCC owing to the highly vascular nature of the tumor. The main regulators of angiogenesis are vascular endothelial growth factor (VEGF), VEGF receptor, and members of the angiopoietin/angiopoietin receptor (Tie-2) families [24]. Hypoxia, acting *via* hypoxia-inducible transcription factors, is a major inducer of angiogenesis [25]. Therefore, VEGF, Tie-2, and the hypoxia-inducible factor 1-α subunit (HIF-1α) are the most potent pro-angiogenic factors, and their over-expression in tumor cells promotes tumor growth in animal models by inducing angiogenesis [26].

Apoptosis, which is a prominent hallmark of cancer [27], is regulated by myeloid cell leukemia-1 (Mcl-1), an anti-apoptotic member of the BCL-2 family proteins, and the cellular inhibitor of apoptosis protein-1 (cIAP-1). Mcl-1 and cIAP-1 are highly expressed in HCC [28], [29]. In addition, apoptosis progresses through activation of the expression of members of the caspase family [30].

In the present study, we employed a diethylnitrosamine (DEN)-induced cancer model that establishes simultaneously HCC and liver cirrhosis [31], since HCC develops from a background of liver cirrhosis induced by repeated inflammation. While this HCC model may be similar to experimental and human HCC, it represents a better tool for studying human HCC than the IR model of HCC without cirrhosis. Previous studies have reported that the intake of 3% BCAA improves IR in mice [15], [32]. BCAA suppresses insulin-induced endothelial cell (EC) tubule formation and liver cancer cell proliferation in a dose-dependent manner [16], [33]. Furthermore, high-dosage BCAA significantly decreases the proliferation of the human (Huh-7) hepatoma cell line, which is not affected by insulin-induced cell proliferation [33]. For this reason, the BCAA dosages of 3% and 6% were used in the present study. Therefore, the aim of the present study was to investigate the anti-cancer and the anti-fibrotic effects of BCAA administration on the development of DEN-induced HCC with liver cirrhosis in a rat model.

## Materials and Methods

### Animals, Reagents, and Diets

Male Sprague–Dawley (SD) rats (approximately 5 weeks old) were obtained from Orient Bio Inc. (Seongnam, Korea). The rats were housed in an air-conditioned room at 25°C with a 12-hr light/dark cycle. All procedure of animal research was provided in accordance with the Laboratory Animals Welfare Act, the Guide for the Care and Use of Laboratory Animals. All animal care and use protocol was reviewed and approved by the Institutional Animal Care and Use Committee (IACUC) in School of Medicine, The Catholic University of Korea. The approval numbers for this study are: CUMC-2010-0033-01, CUMC-2010-0033-02, CUMC-2010-0033-03, and CUMC-2010-0033-04. DEN was purchased from Sigma Chemical Co. (St. Louis, MO, USA). BCAA and casein were obtained from Ajinomoto Co. (Tokyo, Japan). BCAA composition (leucine:isoleucine:valine ratio of 2.0∶1.0∶1.2) was set according to the clinical dosage used for treatment of decompensated liver cirrhosis in Japanese patients [11], [12].

### Experimental Design

The HCC experimental model rats received intraperitoneal injections of DEN at 50 mg/kg body weight once per week for 16 weeks to induce HCC [31], [34]. The rats were divided randomly into three groups beginning 6 weeks after DEN administration (n = 20/group). Rats were fed a diet that contained 3% casein, 3% BCAA, or 6% BCAA for 13 weeks. Five rats in each group were sacrificed to confirm the therapeutic effect of BCAA at 10, 14, 16, and 19 weeks of DEN administration.

### Blood Chemistry Analysis

Blood samples were collected from the inferior vena cava of sacrificed rats at 10, 14, 16, and 19 weeks of DEN administration to measure serum levels of total bilirubin, alanine aminotransferase (ALT), and the plasma level of ammonia. Serum total bilirubin, and ALT were measured using the Biuret method and a kinetic assay, according to the manufacturer’s protocol (BS-400; Mindray, Nanshan, Shenzhen, China). Plasma ammonia was measured using an enzymatic assay (Cobas 6000; Roche, Indianapolis, IN, USA).

### Histopathologic Analysis

The liver was fixed in 10% formalin for 24 hr before being embedded in paraffin. The paraffin-embedded sections were cut to 3- µm thickness and stained with hematoxylin and eosin (H&E). For each case, 10 sections from 10 liver tissue blocks (five each from the right and left lobes) of every group were used to analyze HCC by H&E staining at 16 and 19 weeks of DEN administration. Masson’s trichrome staining (MT) was performed to examine liver collagen deposition at 14 and 16 weeks of DEN administration. The mean areas of dysplastic nodules (DNs) and tumors and the extent of fibrosis in all sections from every group were quantified using a Panoramic MIDI slide scanner and Panoramic viewer software 1.14.50 (3D-Histech Co. Ltd., Budapest, Hungary).

### RNA Extraction and Reverse Transcription

Total RNA was extracted from frozen rat livers using the TRIzol reagent (Invitrogen, Carlsbad, CA, USA), according to the manufacturer’s instructions. Complementary DNA was synthesized from 2 µg total RNA using the oligo-(dT)_12–18_ primer and Superscript III (Invitrogen).

### Semi-quantitative Reverse Transcription-polymerase Chain Reaction (RT-PCR) and Quantitative Real-Time RT-PCR Analysis

Semi-quantitative RT-PCR was carried out with an initial denaturation step at 94°C for 5 min, followed by 20–28 cycles of denaturation at 94°C for 30 s, annealing at 54°–60°C for 30 s, extension at 72°C for 1 min, and a final extension for 5 min at 72°C. Relative band intensity was analyzed using the Gauge 4.0 Image software (Fuji Photo Film Co. Ltd., Tokyo, Japan). Quantitative Real-Time RT-PCR was performed using the Light Cycler® 480 II (Roche) with the Light Cycler® 480 Probes Master Reaction Mix (Roche). The mRNA expression levels were normalized to that of the *glyceraldehyde 3-phosphate dehydrogenase* gene. The sequences of the primers used are shown in Table 1.

**Table 1:** Primer sequences used for semi-quantitative and quantitative Real-Time RT-PCR.

mRNA	Semi-quantitative PCR	Probe No.	Universal Probe Library
	Forward/Reverse primers (5′–3′)		Forward/Reverse primers (5′–3′)
**VEGFA**	TTCAGAGCGGAGAAAGCATT	–	−
	GAGGAGGCTCCTTCCTGC		
**Tie-2**	GCTGAGAACAACATAGGAT	#64	CATAGGAGGAAACCTGTTCACC
	CTGAGTTGAACTGAACAGC		CCCACTTCTGAGCTTCACATC
**HIF-1α**	TCAAGTCAGCAACGTGGAAG	–	−
	TATCGAGGCTGTGTCGACTG		
**TGF-β1**	TGCGCCTGCAGAGATTCAAG	#1	CCTGGAAAGGGCTCAACAC
	AGGTAACGCCAGGAATTGTTGCTA		CAGTTCTTCTCTGTGGAGCTGA
**Col1α2**	GCTGAGGGCAACAGCAGATTC	#64	ACGGTCTGGATGGATTGAAA
	GATGTCCAGAGGTGCAATGTCAA		GACCTGGGGTTCCATTCTCT
**Col3α1**	TGGACAGATGCTGGTGCTGAG	#79	GTGAACCGGGTCAAGCTG
	GAAGGCCAGCTGTACATCAAGGA		GGGCCAGATGGACCAATA
**Smad-4**	GTTGCAGATAGCTTCAGGGC	#10	GAACACTGGATGGACGACTG
	GGATCCACGTATCCATCCAC		TGTTTTAGTTCGTTCTTGTGTAGATCA
**TIMP-1**	TCCCCAGAAATCATCGAGAC	–	–
	TCAGATTATGCCAGGGAACC		
**TIMP-2**	CAAGTTCTTTGCCTGCATCA	#10	CGTTTTGCAATGCAGACGTA
	TCCAGGAAGGGATGTCAAAG		GATGGGGTTGCCATAGATGT
**Mcl-1**	TGGACATTAAAAACGAGGACG	–	–
	AAGAACTCCACAAACCCATCC		
**cIAP-1**	CGAGGAGGAGGAGTCAGA	#81	CCCAGAGGATGAGACTGGAG
	GCACTTAGGAGGCAATCCAG		TCACTGCATCTTCCCAATTCT
**GAPDH**	AGACAGCCGCATCTTCTTGT	Universal Probe Library Rat *GAPD* geneassay probe	Universal Probe Library Rat *GAPD*gene assay primer
	CTTGCCGTGGGTAGAGTCAT		

### Protein Extraction and Western Blotting

Liver tissues were homogenized and equivalent amounts of protein lysates (10 µg/lane) were separated by 12% sodium dodecyl sulfate-polyacrylamide gel electrophoresis and subjected to Western blotting using primary antibodies directed against phosphorylated β-catenin (p-β-catenin) (Thr41/Ser45), phosphorylated p38 mitogen-activated protein kinase (p-p38 MAPK [Thr180/Tyr182]), caspase-3 (Cell Signaling Technology, Danvers, MA, USA), β-catenin (Enzo Life Sciences, Farmingdale, NY, USA), p38 MAPK, VEGF (Santa Cruz Biotechnology, Santa Cruz, CA, USA), proliferating cell nuclear antigen (PCNA; DAKO, Carpentaria, CA, USA), tissue inhibitor of metalloproteinase-2 (TIMP-2; Abcam, Cambridge, MA, USA), and α-smooth muscle actin (α-SMA; Sigma-Aldrich, St. Louis, MO, USA). An antibody against β-actin (Sigma-Aldrich) served as a loading control. Relative blot intensity was quantified with the Gauge Image software, ver. 4.0.

### Statistical Analysis

The results for the serum and plasma assays, MT staining, semi-quantitative and quantitative Real-Time RT-PCR, Western blotting, and tumor area and number measurements are expressed as means ± standard deviations. The statistical significance of differences between the groups was determined using the Student’s *t-*test. A value of *P*<0.05 was considered to indicate statistical significance.

## Results

### Development of HCC Following DEN Administration

**Figure 1:**
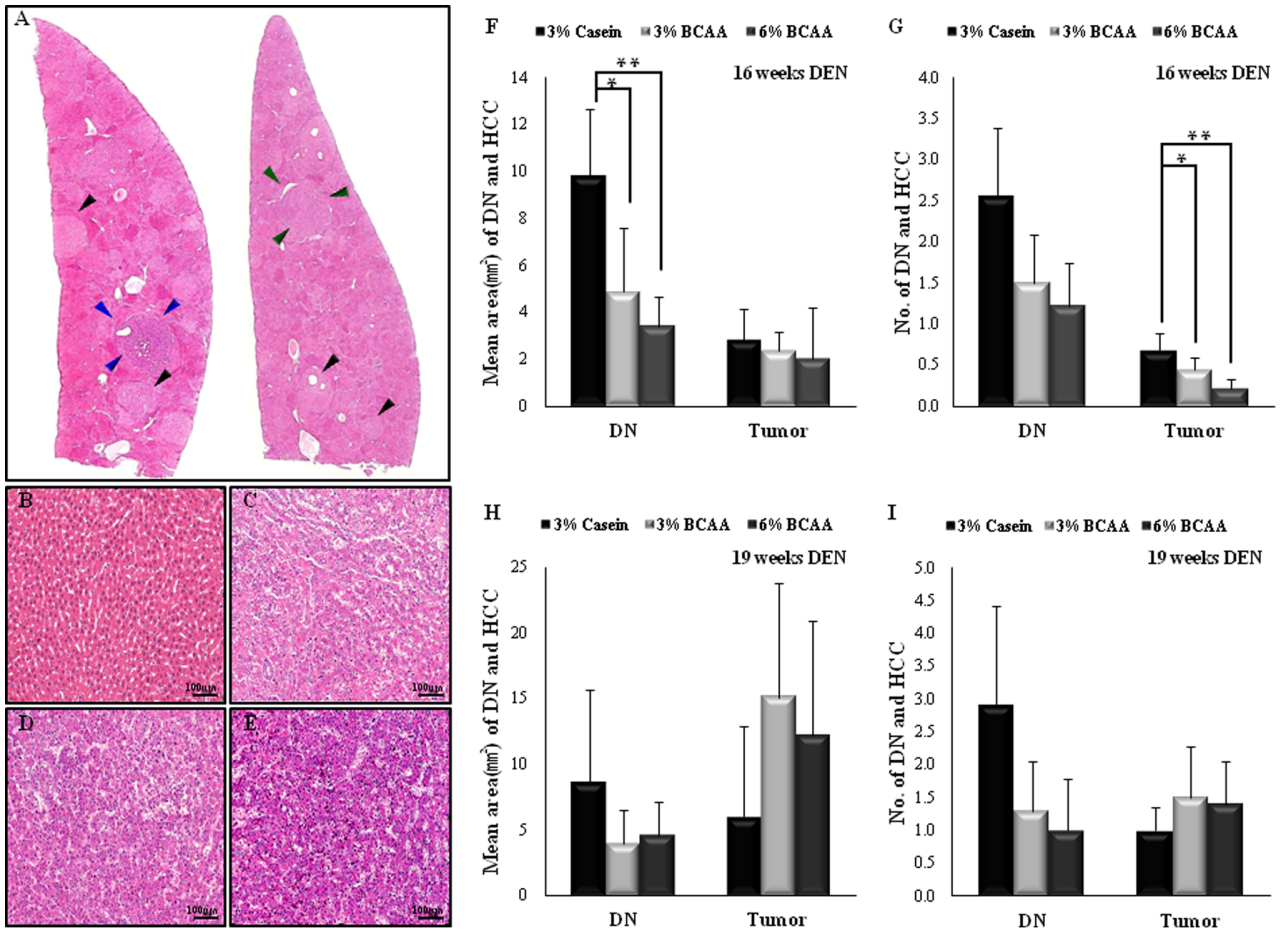
Microscopic analyses of DEN-induced HCC with liver cirrhosis. Pathologic findings after 16 weeks of DEN administration; hematoxylin and eosin (H&E) staining. DN, dysplastic nodule, black arrowheads; early HCC, green arrowheads; HCC, blue arrowheads; A, original magnification ×5; B, normal; C, DN; D, early HCC; E, HCC; B–E, original magnification ×200. The mean areas (F and H) and numbers (G and I) of DNs and tumors after 16 and 19 weeks of DEN administration were evaluated and compared. Values shown are means ± standard deviation (n = 5/group). *P<0.05; **P<0.005.

No significant differences in body or liver weights were observed among the groups (Table 2). The 6% BCAA group revealed a significantly decreased liver/body weight ratio compared with the casein group (*P*<0.05), indicating reduced tumor burden following BCAA treatment. Sequential development of cirrhosis and HCC were identified in livers of experimental rats treated with DEN for 10 weeks. As shown in Figure 1, we evaluated the area and number of gross intrahepatic tumors after 16 and 19 weeks of DEN administration (Fig. 1A). Representative histopathologic photomicrographs of a normal liver (Fig. 1B), DN (Fig. 1C), early HCC (Fig. 1D), and HCC (Fig. 1E) in the livers of experimental rats are shown.

**Table 2:** Liver/body weight ratio and results of biochemical analyses according to the duration of DEN administration in each group.

At 10 weeks of DEN administration										
Diet	n	Body weight (g)		Liver weight (g)		Liver/body weight (%)		ALT (U/L)	T.bilirubin (mg/dl)	Ammonia (µg/dl)
**3% Casein**	5	427±31.1		11±1.7		2.6±0.4		59.2±13.48	0.16±0.05	211.8±55.94
**3% BCAA**	5	420±29.8		9.9±1.2		2.3±0.2		52.6±7.3	0.1±0.00	193.6±23.92
**6% BCAA**	5	417±20.2		10.3±1.6		2.5±0.3		70±15.59	0.16±0.05	247.25±17.27
**At 14 weeks of DEN administration**										
**Diet**	**n**	**Body weight (g)**	**Liver weight (g)**		**Liver/body weight (%)**		**ALT (U/L)**		**T.bilirubin (mg/dl)**	**Ammonia ( µg/dl)**
**3% Casein**	5	463.9±42.3	22.1±5.2		4.8±1.1		126.8±70.46		0.42±0.18	448.4±68.96
**3% BCAA**	5	464.4±39.6	20.1±4.9		4.3±0.8		105.6±49.45		0.32±0.16	**347.2±80.01** *
**6% BCAA**	5	450±30.2	19.2±3.2		4.3±0.9		109.6±29.67		0.38±0.13	**244±45.73** ***
**At 16 weeks of DEN administration**										
**Diet**	**n**	**Body weight (g)**	**Liver weight (g)**		**Liver/body weight (%)**		**ALT (U/L)**		**T.bilirubin (mg/dl)**	**Ammonia ( µg/dl)**
**3% Casein**	5	484.7±51.6	27±3.8		5.6±1.0		152.2±61.73		1.45±0.65	415.8±66.27
**3% BCAA**	5	464.6±36.3	25.6±2.2		5.5±0.8		117±34.63		**0.76±0.22** *	361±36.57
**6% BCAA**	5	457.4±43.7	20.7±3.1		**4.5±0.7** *		134.6±23.95		**0.6±0.37** *	**274.6±32.99** **
**At 19 weeks of DEN administration**										
**Diet**	**n**	**Body weight (g)**	**Liver weight (g)**		**Liver/body weight (%)**		**ALT (U/L)**		**T.bilirubin (mg/dl)**	**Ammonia ( µg/dl)**
**3% Casein**	5	494.9±34.1	25.2±3.7		5.1±1.0		122.4±37.75		0.24±0.05	368.6±56.56
**3% BCAA**	5	515.1±26.2	33.1±7.7		6.5±1.7		170±32.52		0.25±0.06	349.4±28.39
**6% BCAA**	5	504.4±51.9	31.1±8.7		6.2±1.6		119.2±12.66		0.4±0.16	**282.8±30.7** *

Values shown are means ± SD.

* Significantly different from the 3% casein group at *P*<0.05.

** Significantly different from the 3% casein group at *P*<0.005.

*** Significantly different from the 3% casein group at *P*<0.0005.

### Effects of BCAA on Liver Functions of DEN-treated Rats

As shown in Table 2, the BCAA groups tended to have decreased serum levels of ALT, as compared with the casein group. Serum total bilirubin levels were decreased markedly in the BCAA groups, as compared with the casein group after 16 weeks of DEN administration (3% and 6% BCAA; *P*<0.05), indicating improved liver function. In addition, the plasma ammonia levels in the BCAA groups were significantly lower than those in the casein group after 14 weeks (3% BCAA; *P*<0.05, 6% BCAA; *P*<0.0005), 16 weeks (6% BCAA; *P*<0.005), and 19 weeks (6% BCAA; *P*<0.05) of DEN administration. The Homeostasis Model Assessment of Insulin Resistance resulted gave a score <3, suggesting that this experimental model is not an IR rat model after 10, 14, and 16 weeks of DEN administration, although it is after 19 weeks of DEN administration (data not shown).

### Effects of BCAA on Hepatocarcinogenesis in DEN-treated Rats

Both the mean area and the number of DNs and tumors in the casein group tended to be larger than those in the BCAA groups (Fig. 1F, G). In particular, the mean DN area decreased significantly in the BCAA groups (3% BCAA; *P*<0.05, 6% BCAA; *P*<0.005) compared with the casein group. The BCAA groups also had significantly fewer tumors compared with the casein group (3% BCAA; *P*<0.05, 6% BCAA; *P*<0.005). However, no differences were observed for the mean areas or numbers of DNs and tumors after 19 weeks of DEN administration among the three groups (Fig. 1H, I).

### Effects of BCAA on Fibrosis of the Livers of DEN-treated Rats

**Figure 2:**
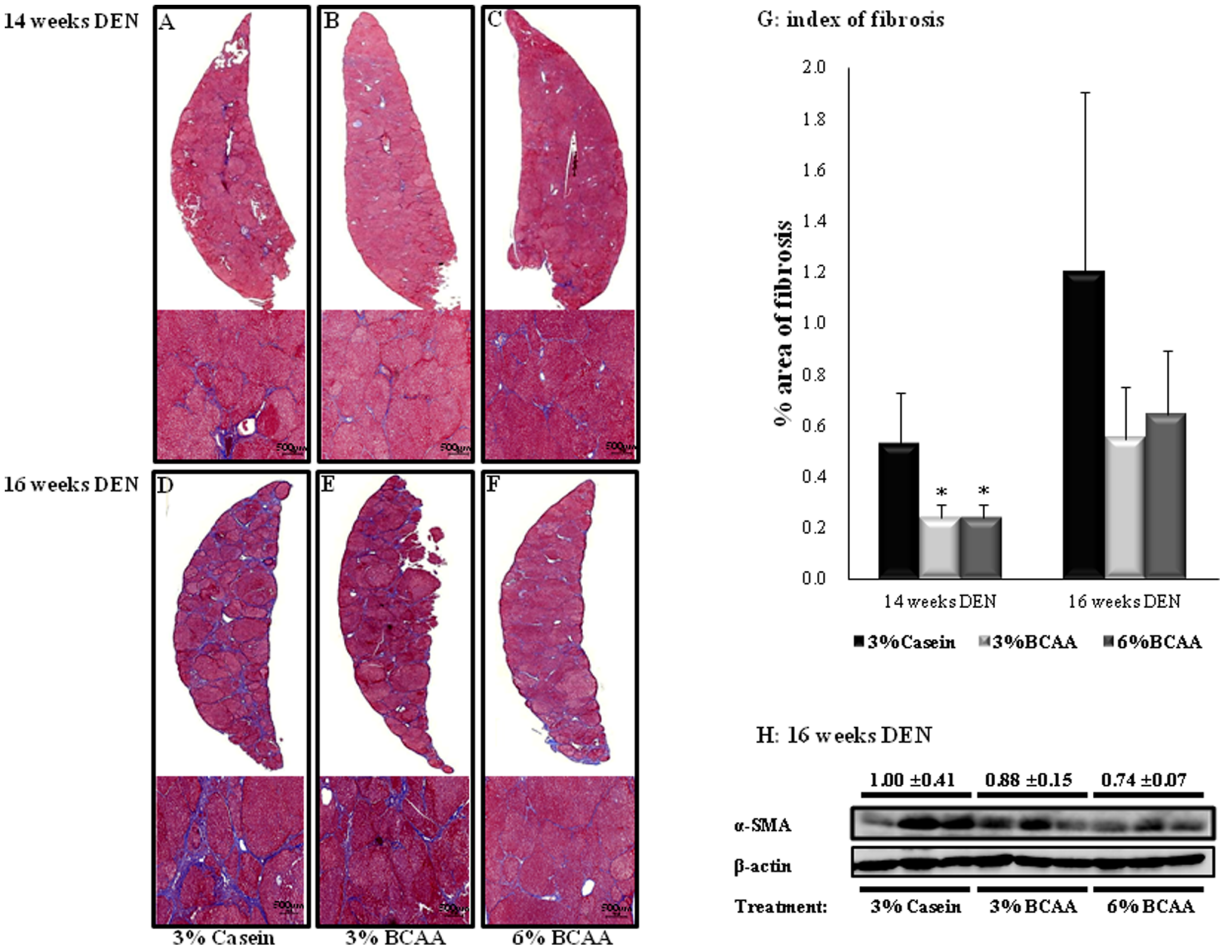
Effects of BCAA on DEN-induced liver fibrosis. Paraffin-embedded sections were stained with Masson’s trichrome to evaluate the progression of fibrosis after 14 and 16 weeks of DEN administration (A–F, original magnification ×5 and ×50). Index of fibrosis (G). The expression of α-smooth muscle actin (SMA) protein in livers was evaluated by Western blot analysis at 16 weeks post-DEN injection (H). An anti-β-actin antibody served as the loading control. Values shown are means ± standard deviation (n = 5/group). *P<0.05. Relative fibrosis area: rMA = (MA/FA)×100. FA, overall field area (mm^2^); MA, overall mask area (mm^2^), which is the summed areas for each detected object in each layer.

**Figure 3:**
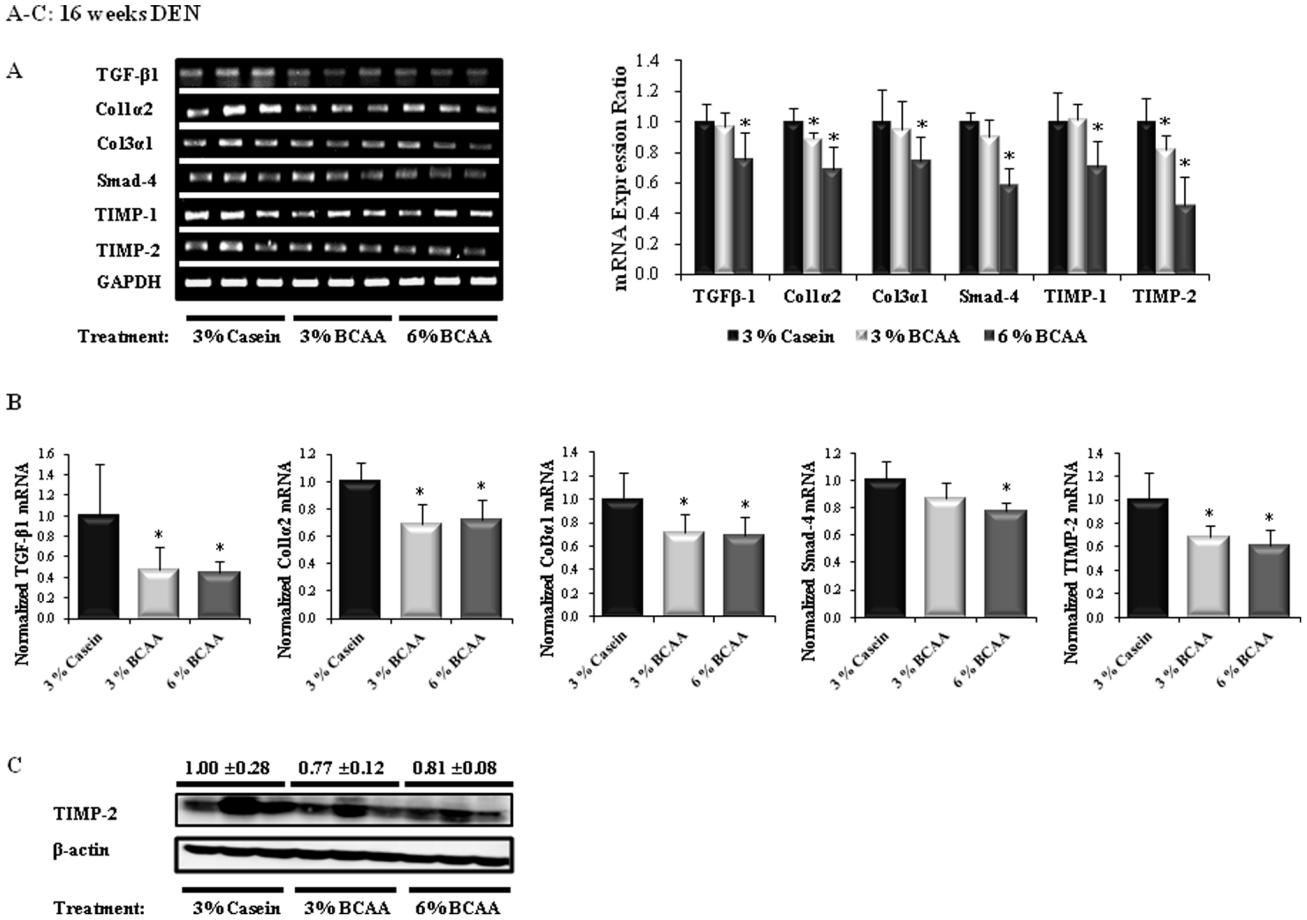
Effects of BCAA supplementation on liver fibrosis in rats after 16 weeks of DEN administration. The relative band intensities of the fibrosis markers are shown in the right-hand panels (A). The expression levels of mRNA for TGF-β1, Col1α2, Col3α1, Smad-4, and TIMP-2 were determined by quantitative Real-Time reverse-transcription polymerase chain reaction (RT-PCR) (B). TIMP-2 protein expression was determined by Western blotting (C). The level of expression of each gene is normalized to those of GAPDH (A and B) and β-actin (C). The lanes contain the mRNA (A) and protein (C) samples from three rats per group. Values shown are means ± standard deviation. *P<0.05.

The mean fibrotic area in the BCAA groups was lower than that in the casein group after 14 and 16 weeks of DEN administration, based on MT staining (Fig. 2A–F). Liver fibrosis decreased significantly at 14 weeks in the BCAA groups (Fig. 2A–C; 3% and 6% BCAA; *P*<0.05). Furthermore, BCAA improved liver fibrosis compared to that in the casein group at 16 weeks (Fig. 2D–F). A quantitative analysis also showed that the mean fibrotic areas of the BCAA groups were lower than those of the casein group at 14 and 16 weeks (Fig. 2G). In addition, Western blot analyses showed lower α-SMA protein expression in the BCAA groups than in the casein group at 16 weeks (Fig. 2H). Furthermore, the BCAA groups had downregulated expression of mRNA for fibrosis markers (TGF-β1, Col1α2, Col3α1, TIMP-1, and TIMP-2) at 16 weeks of DEN administration, as compared with the corresponding levels in the casein group. Significant lower levels of mRNA for TGF-β1 (6% BCAA; *P*<0.05), Col1α2 (3% and 6% BCAA; *P*<0.05), Col3α1, Smad-4, TIMP-1 (6% BCAA; *P*<0.05), and TIMP-2 (3% and 6% BCAA; *P*<0.05) were observed in the BCAA groups than in the casein group (Fig. 3A). Similarly, quantitative Real-Time RT-PCR showed that the BCAA treatment resulted in significant decreases in the levels of mRNA for TGF-β1, Col1α2, Col3α1 (3% and 6% BCAA; *P*<0.05), Smad-4 (6% BCAA; *P*<0.05), and TIMP-2 (3% and 6% BCAA; *P*<0.05), as compared with the casein group (Fig. 3B). In addition, Western blot analyses showed lower TIMP-2 protein expression in the BCAA groups than in the casein group at 16 weeks (Fig. 3C).

### Effects of BCAA on Angiogenesis and Apoptosis in the Livers of DEN-treated Rats

**Figure 4:**
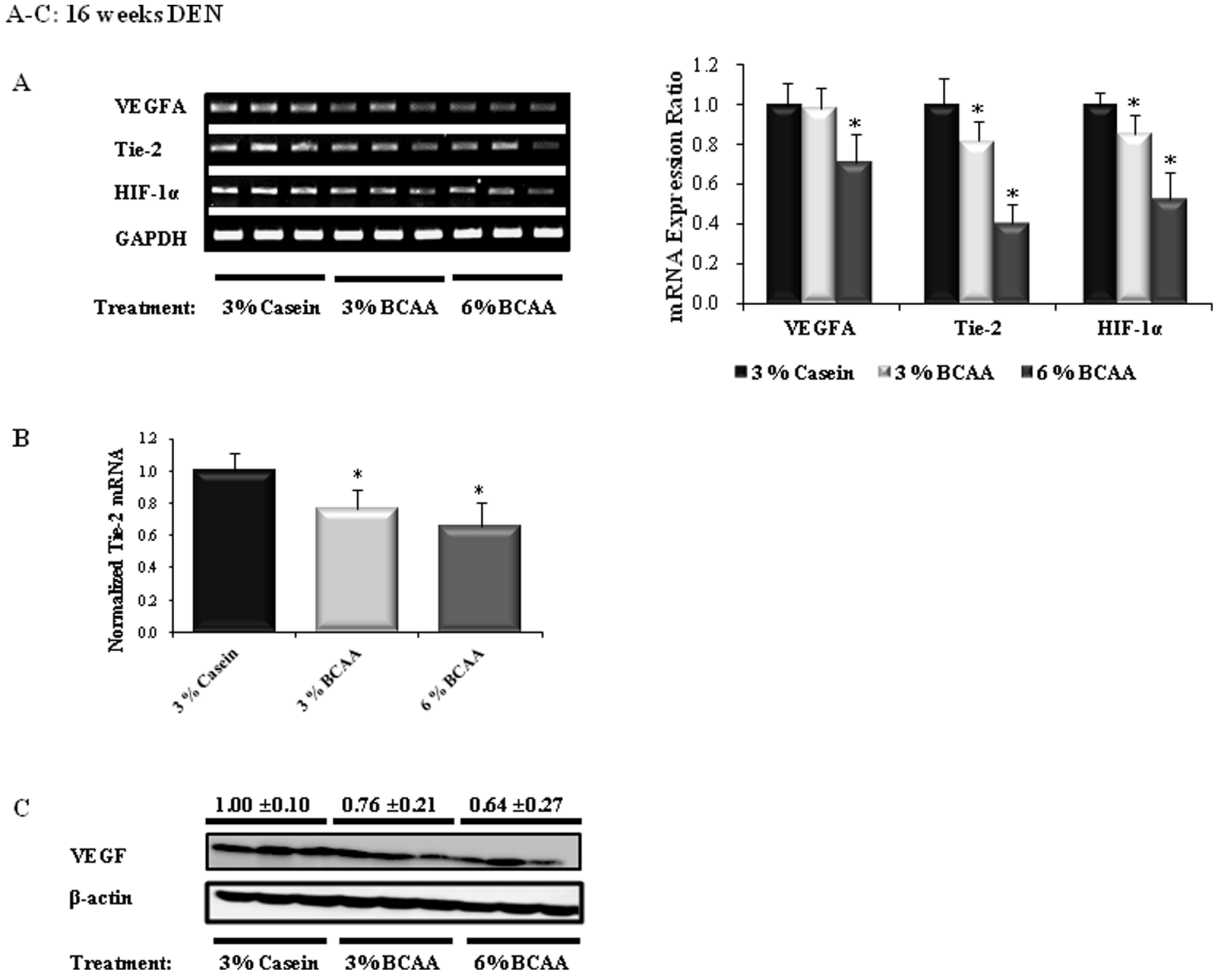
Effects of BCAA supplementation on angiogenesis in rats after 16 weeks of DEN administration. The relative band intensities of the angiogenesis markers are shown in the right-hand panels (A). The expression level of Tie-2 mRNA was determined by RT-PCR (B). VEGF protein expression was determined by Western blotting (C). The expression level of each gene is normalized to those of GAPDH (A and B) and β-actin (C). The lanes contain mRNA (A) and protein (C) samples from three rats per group. Values shown are means ± standard deviation. *P<0.05.

**Figure 5:**
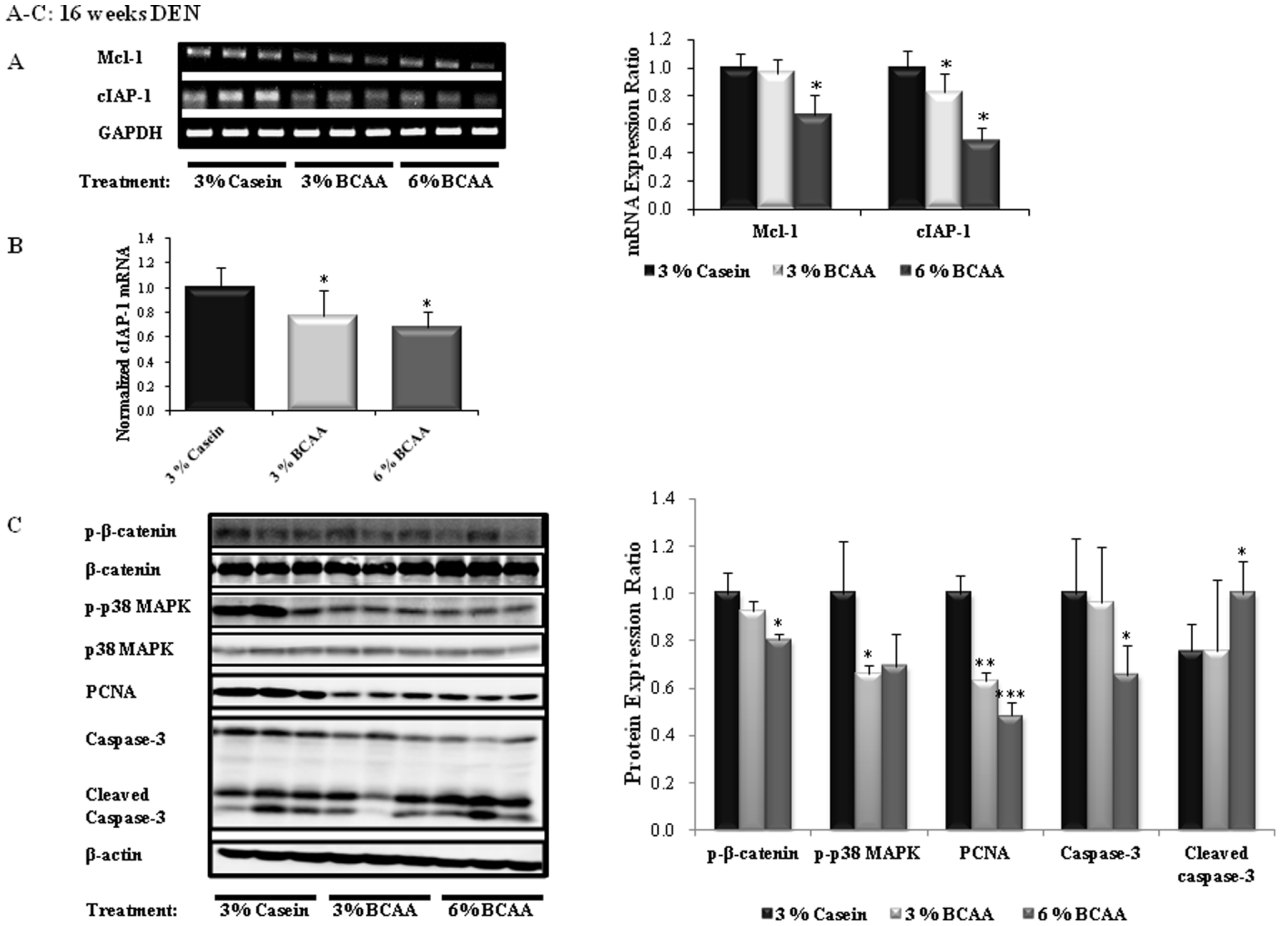
Effects of BCAA supplementation on apoptosis in rats after 16 weeks of DEN administration. The relative band intensities of the apoptosis inhibitor markers are shown in the right-hand panels (A). The mRNA expression levels of Mcl-1 and cIAP-1 were determined by RT-PCR (B). The expression levels of p-β-catenin, p-p38 MAPK, PCNA, and caspase-3 protein were determined by Western blotting (C). The expression level of each gene is normalized to those of GAPDH (A and B) and β-actin (C). The lanes contain mRNA (A) and protein (C) samples from three rats per group. Values shown are means ± standard deviation. *P<0.05; **P<0.005; ***P<0.0005.

The BCAA groups showed downregulated expression of mRNA for angiogenesis markers (VEGF, Tie-2, HIF-1α) and apoptosis inhibitor markers (Mcl-1, cIAP-1) at 16 weeks of DEN administration, as compared to the corresponding levels in the casein group. Significant decreases in the levels of mRNA for VEGFA (6% BCAA; *P*<0.05), Tie-2, HIF-1α (3% and 6% BCAA; *P*<0.05), Mcl-1 (6% BCAA; *P*<0.05), and cIAP-1 (3% and 6% BCAA; *P*<0.05) were observed in the BCAA groups, as compared to the casein group (Figs. 4A and 5A). Similarly, quantitative Real-Time RT-PCR showed that BCAA treatment resulted in significant decreases in the levels of mRNA for Tie-2 and cIAP-1 (3% and 6% BCAA; *P*<0.05), as compared to the casein group (Figs. 4B and 5B).

As shown in Figures 4C and 5C, the BCAA groups exhibited markedly decreased expression of VEGF, p-β-catenin, p-p38 MAPK, PCNA, and caspase-3 proteins and increased levels of cleaved caspase-3 protein, as compared to the casein group after 16 weeks of DEN administration. Western blot analysis revealed significant decreases in the levels of p-β-catenin (6% BCAA; *P*<0.05), p-p38 MAPK (3% BCAA; *P*<0.05), PCNA (3% BCAA; *P*<0.005, 6% BCAA; *P*<0.0005), and caspase-3 (6% BCAA; *P*<0.05) and increased expression of cleaved caspase-3 (6% BCAA; *P*<0.05).

## Discussion

Hyperammonemia develops due to failure of ammonia detoxification during the progression of chronic liver disease or portal-systemic shunting [35], [36]. BCAA administration decreases blood levels of ammonia through a catabolic effect that influences glutamate dehydrogenase activity and reduces the rate of glutamate breakdown [36], [37]. In the present study, the plasma levels of ammonia in the BCAA groups were significantly lower than those in the casein group after 14, 16, and 19 weeks of DEN administration. The BCAA groups also tended to have lower levels of serum ALT and significantly lower levels of total bilirubin, as compared with those in the casein group, suggesting an improvement in the liver damage associated with BCAA treatment.

The present study is distinguished from recent studies in that we investigated fully differentiated HCC following chemical induction through a stepwise process, i.e., fibrosis, DN, and HCC. We demonstrate that BCAA reduces both premalignant DN and HCC development in the model after 16 weeks of therapy. Furthermore, liver fibrogenesis was prevented by BCAA supplementation.

Our results show inhibition of the development of liver neoplasms and reductions in the sizes of preneoplastic lesions. However, no differences with respect to the mean areas or numbers of DNs or tumors were observed at 19 weeks of DEN administration among the three groups. Therefore, BCAA treatment longer than 19 weeks does not prevent the progression of malignant tumors, since BCAA is not a chemotherapeutic agent and may be inadequate for the treatment of advanced HCC.

Recent studies have determined that all *Smad* genes are critical downstream mediators of TGF-β1 and Col1α1, and that Col3α1 and TIMP-1 are new *Smad3/4* gene targets [38]. That study also reported that TIMP-1 and TIMP-2 are crucial factors for promoting the progression of liver fibrosis [19], [39]. Nakanishi et al. have shown that valine treatment ameliorates liver fibrosis in a rat model induced with carbon tetrachloride [40]. In the present study, the mean fibrotic area decreased in the BCAA groups after 14 and 16 weeks of DEN administration; the level of α-SMA protein expression also decreased in the BCAA groups, as compared to the casein group after 16 weeks of DEN administration. In addition, we demonstrate that BCAA significantly decreases the expression of mRNA for fibrosis markers (TGF-β1, Col1α2, Col3α1, TIMP-1, TIMP-2), as compared to those in the casein group after 16 weeks of DEN administration. Western blotting showed a significant decrease in TIMP-2 expression after 16 weeks of DEN administration. Interestingly, we found that 6% BCAA had greater anti-fibrotic effect than 3% BCAA in the DEN-induced cirrhosis model.

The primary mechanism of BCAA is the inhibition of tumor angiogenesis [41]. The induction of VEGF by HIF-1α, which is the onset of the angiogenic switch, involves the MAPK pathway in HCC cells [42]. Our results consistently show that the expression levels of VEGF, HIF-1α, and p-p38 MAPK are decreased by BCAA treatment. In addition, Tie-2 was down-regulated by BCAA therapy. Interestingly, BCAA supplementation prevented effective tumor growth by suppressing angiogenesis during the early stage of HCC (at 16 weeks), whereas it did not prevent angiogenesis in the advanced stage of HCC (at 19 weeks).

Hagiwara *et al*. have reported that Huh-7 cell proliferation is not affected by insulin, although cell proliferation decreases significantly to a level less than that observed in the absence of insulin following high-dose BCAA treatment [33]. In the present study, PCNA protein expression decreased through the suppressive effect of BCAA on p38 MAPK and β-catenin phosphorylation. Furthermore, BCAA treatment significantly decreased the expression of mRNA for apoptosis inhibitor markers (Mcl-1, cIAP-1) and it activated caspase-3 protein expression, as compared to the casein group, which supports the concept that BCAA suppresses cell proliferation by inducing the apoptosis of liver cancer cells. Therefore, we suggest that 6% BCAA has a more potent chemopreventive effect than 3% BCAA in the DEN-induced HCC with cirrhosis model, but not in the IR model.

Based on our results, we suggest that BCAA improves liver fibrosis by downregulating Smad-4, TIMP-1, and Col1α2 through the inhibition of TGF-β1. Furthermore, BCAA supplementation suppresses HCC angiogenesis and cell proliferation and increases the apoptosis of liver cancer cells.

In conclusion, BCAA supplementation in the diet improved liver fibrosis and prevented the development of HCC in a DEN-induced rat model of HCC with liver cirrhosis. These results may provide a rationale for the use of BCAA treatment as an anti-fibrotic and chemopreventive therapy against HCC with liver cirrhosis.
